# Bacterial meningitis in adults: therapeutic challenges in the era of antibiotic resistance and the potential of bacteriophages and associated by products

**DOI:** 10.3389/fcimb.2026.1755353

**Published:** 2026-02-06

**Authors:** Magdalena Dzięgiel, Zuzanna Głodowicz, Aleksandra Jóźwiak, Weronika Roztkowska, Agnieszka Necel, Lidia Piechowicz

**Affiliations:** 1 Student Scientific Circle of Medical Microbiology, Department of Medical Microbiology, Faculty of Medicine, Medical University of Gdańsk, Gdańsk, Poland; 2 Department of Medical Microbiology, Faculty of Medicine, Medical University of Gdańsk, Gdańsk, Poland

**Keywords:** antibiotic resistance, antibiotic therapy, meningitis, meningitis-related bacteria, phage therapy

## Abstract

Bacterial resistance to antibiotics is one of the leading factors encouraging the development of new therapeutic strategies. The increased resistance to antibiotics can be attributed to several factors, such as early and unnecessary administration, incorrect dosing, or incomplete antibiotic treatment. One of the diseases that calls for improved understanding of this problem is meningitis, which - if ineffectively treated - may result in severe neurological complications and death. This study provides an overview of the current antibiotic strategies for bacterial meningitis along with the therapeutic challenges associated with standard treatment options. In addition, it also presents the current progress in bacteriophage research, highlighting both their potential to replace some common antibiotic therapies in the treatment of meningitis and the significant lack of clinical studies regarding most of them. The research on phage therapy targeting meningitis-associated pathogens is limited, and where it exists, it is predominantly focused on mouse models. There, its efficiency seems mostly promising. Nevertheless, comprehensive clinical trials are needed to properly determine the efficacy and safety of phage therapy in humans before it becomes a significant alternative to antibiotics.

## 1 Introduction

It is undeniable that antibiotics revolutionized the world of bacterial infectious diseases. Unfortunately, the continuing rise of antibiotic resistance among the most common pathogens, along with a considerable decrease in the production of new antibiotics, could result in a global health crisis in the future (Hutchings et al., 2019; Muteeb et al., 2023). That is why alternative antimicrobial agents are explored, or **-** to be more precise in the case of bacteriophage therapy**-** revisited.

Phage therapy has been around for years. Félix d’Herelle described the “anti-Shiga microbe” in 1917, which - thanks to his further experimental studies - proved to be an obligate bacteriophage effective against the Shiga bacilli (Wittebole et al., 2014). Due to the introduction of sulfa drugs and penicillin to treatment (1930 and 1940, respectively), the bacteriophage research was greatly overshadowed by the growing interest in the antibiotics. As a result, in many Western countries phage therapy was relegated to history. Bacteriophages remained actively studied only in the former USSR, Poland, and India (Wittebole et al., 2014). However, increasingly common reports on multidrug-resistant (MDR) and extensively drug-resistant (XDR) strains have led researchers to explore possible alternatives to antibiotics **-** and so, phage therapy has gained its spotlight yet again.

Meningitis is a life-threatening condition caused by various infectious and non-infectious processes. This study focuses specifically on bacterial meningitis, which occurs when a bacterial infection spreads to cerebrospinal fluid (CSF) through hematogenous seeding or direct contiguous spread (from anatomically adjacent structures, foreign bodies, or during neurosurgical procedures). However, it also happens as a result of primary infections like pneumonia or even burn wounds, which implies that meningitis can be a serious complication in most types of infection (Winkelman and Galloway, 1992; Marra and Brigham, 2001; Calvano et al., 2010; Perez and Haith, 2017; Lourens et al., 2025). Bacteria, their toxins, and their metabolites irritate the surrounding tissue, thus causing the inflammation of the dura mater, arachnoid mater, and pia mater. According to the World Health Organization, 1.6 million cases of bacterial meningitis were reported in 2019, 240,000 of which were fatal. It is estimated that 1 out of 6 patients affected by bacterial meningitis will die, and 1 out of 5 will experience neurological deficits, limb amputation, skin scarring, or other long-lasting disabilities as a result of this disease (WHO, 2024).

**Figure 1:**
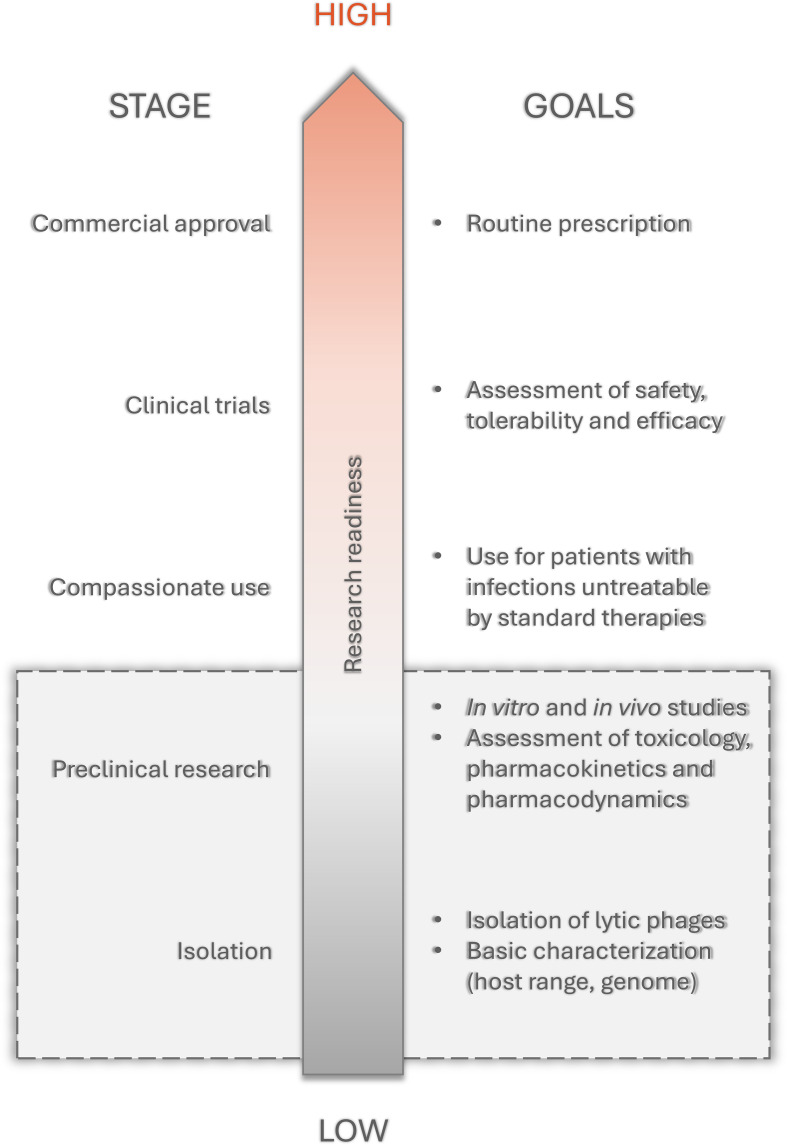
The phage therapy research readiness scheme. For meningitis most research is focused on stages in gray box.

The deadliness and sequelae of meningitis treated in accordance with the current recommendations, along with the growing antibiotic resistance, encouraged scientists to revisit the use of alternative antibacterial therapies such as phage therapy. This review aims to present the current state of knowledge on phages in the treatment of bacterial meningitis in adults to help navigate through this vast area of research and point to niches that need further exploration. Adults between 24 and 65 years old are the group with the least rate of meningitis, so most research is focusing on infection of the higher-risk groups like children or elders. Therefore, we decided to focus on that group to prevent it from being marginalized and to point out the rare incidents occurring in that group. This paper also attempts to outline the possible obstacles in the phage treatment of meningitis as well as its advantages and disadvantages compared to standard antibiotic therapy. Papers included in this manuscript were searched through the NCBI database with a focus on the last 20 years and a main emphasis on the years 2015-2025. At first, experiments aiming to use phages directly in bacterial meningitis treatment were collected. If there was no direct application to treat the disease, the gaps were filled with research focusing on the use of phages or their products on the chosen etiological factors *in vitro* or on animal models (mostly mice) representing the most common primary infections. Although such data may not adequately show the phages’ ability to fight meningitis (**Figure 1**), it might encourage other scientists to try these viruses also in central nervous system infections.

## 2 Clinical and laboratory differentiation of meningitis

The clinical features aren’t specific enough to be able to differentiate between bacterial and viral meningitis with satisfactory certainty. Symptoms like fever, headache, nuchal rigidity, and altered consciousness aren’t only specific to meningitis, let alone its specific subtypes. Nevertheless, the presence of two or more of those symptoms along with other meningeal signs (Kernig’s sign, Brudzinski’s sign) is a definite indication that a lumbar puncture (LP) is needed to rule out or confirm meningitis [5]. The composition of cerebrospinal fluid, along with an adequate microbiological examination (Gram stain and culture, serological tests), should be assessed to properly make a diagnosis. For example, turbid or cloudy CSF can only be observed in bacterial meningitis (**Table 1**). PCR tests can also be performed on the CSF to help quickly identify the specific pathogen, particularly in cases where traditional cultures may be inconclusive or slow to yield results. With a high index of suspicion of bacterial meningitis, empirical antibiotics can be started after the lumbar puncture is undertaken. However, LP is still needed both to fully diagnose and to confirm the effectiveness of the antibiotic therapy (Hrishi and Sethuraman, 2019). It is also worth noting that if mycobacterial or nocardial meningitis etiology is suspected, additional microbiological tests are needed to differentiate between them due to their acid-fastness (Muricy et al., 2014).

**Table 1:** CSF composition in healthy and infected individuals (↑ - increased level, ↓ - lowered level; number of arrows indicates the strength of the effect) (Hrishi and Sethuraman, 2019; Carter and McGill, 2022).

Parameter	Standard CSF	Bacterial meningitis	Tuberculous meningitis	Viral meningitis
Opening pressure (cm H_2_O)	12-20	↑↑↑	↑↑↑	Normal/↑
Colour	Clear	Turbid, cloudy	Clear/cloudy	Clear
White cell count	<5 cells/μL	↑ (>100 cells/μL)	↑↑ (5–500 cells/μL)	↑↑↑ (5–1000 cells/μL)
Predominant cell type	Lymphocytes	Neutrophils	Lymphocytes	Lymphocytes
Glucose level	50–80 mg/dL	↓↓↓	↓↓↓	Normal/↓
CSF: Plasma glucose ratio	>0,66	↓↓↓	↓↓↓	Normal/↓
Protein level	15–40 mg/dL	↑↑	↑↑↑	Normal

Although the clinical signs aren’t characteristic for specific species of bacteria, there are certain things in the clinical picture that may hint at the most probable groups of species. When it comes to postoperative meningitis, specifically post-neurosurgical meningitis (e.g., post craniectomy), CNS *Staphylococci*, *Staphylococcus aureus*, and *Cutibacterium acnes* infections should be suspected. If those three options are ruled out, one has to remember that aerobic gram-negative bacilli can also be the possible etiological factors of post-neurosurgical meningitis (Hussein et al., 2017). Additional risk groups should also be investigated. For instance, the newborns, the pregnant women, the elderly, and the immunocompromised are more likely to be at risk of *Listeria monocytogenes* infections and meningitis (Pagliano et al., 2016). Patients with a removed or damaged spleen are at risk of developing meningitis caused by *Streptococcus pneumoniae, Haemophilus influenzae*, or *Neisseria meningitidis*, while meningitis in alcoholics is often associated with infection by strains of *S. pneumoniae* or *L. monocytogenes* (Roos et al., 1997; Albrecht, 2011).

In adults, the most common bacteria causing bacterial meningitis are *S. pneumoniae, N. meningitidis*, *H. influenzae* (mainly type b, Hib), and *L. monocytogenes* (Brouwer et al., 2010). Apart from the factors mentioned above, under certain clinical conditions, bacteria like, for example, *Mycobacterium tuberculosis* or *Klebsiella pneumoniae* may also occur (Marx and Chan, 2011). Unfortunately, establishing the right etiology of meningitis is still a complicated task that takes a lot of time and effort. When extreme antibiotic resistance is added to that equation, there is usually not a lot of time to change antibiotics and make sure that the therapy is fully adequate. That’s why phage therapy of meningitis should be given more consideration. It should be emphasized that most bacterial strains don’t yet have resistance against bacteriophages, which eliminates the additional time needed to establish the right antibiotic therapy. Along with the promising *in vitro* efficiency of phage therapy, this might lead to a higher success rate of restoring meningitis patients to health and limiting the neurological sequelae in the future.

## 3 Antibiotic resistance and therapy against meningitis-associated pathogens in adults

The discovery of penicillin in the first half of the 20th century completely changed the world of medicine. Since then, many new classes of antibiotics have been identified and implemented in clinical practice. Because the type of antibiotic applied in therapy strongly depends on the etiological factor and its intrinsic/acquired resistance, it is important to observe the changes in bacterial characteristics and continuously update recommendations. Therefore, the following information represents both antibiotic resistance and the therapies used in most countries, based on the clinical cases and general recommendations from all over the world.

*Neisseria meningitidis* is a Gram-negative bacterium that is one of the major causes of rapidly progressing bacterial meningitis in humans worldwide. It colonizes the nasopharynx (carrier) and spreads mostly *via* large respiratory droplets. A carrier of *N. meningitidis* is the main source of infection. Additionally, the carriage of *N. meningitidis* may persist even for 5–6 months (Mikucki et al., 2022). Due to the rapid course of the disease, treatment should be initiated as soon as possible – even before the etiology is confirmed. In empirical treatment, ceftriaxone (or cefotaxime) – β-lactam antibiotics with a broad spectrum of activity – are effective against *N. meningitidis*. Sometimes vancomycin is added in case of suspected *S. pneumoniae* meningitis resistant to β-lactams. If the presence of *N. meningitidis* is confirmed and its susceptibility is known, penicillin G is traditionally considered the drug of choice for susceptible strains. Ceftriaxone is also still recommended, especially if susceptibility testing has not been performed (Albrecht, 2011).

The reduced susceptibility of *N. meningitidis* to antibiotics used in therapy is a rare phenomenon. *N. meningitidis* mostly remains sensitive to penicillin and third-generation cephalosporins. However, isolated cases of strains with reduced susceptibility to penicillin have been reported, mainly due to the formation of altered PBP2 protein and production of β-lactamase (Stefanelli et al., 2003). Although reduced susceptibility (elevated MIC, but not resistance) of *N. meningitidis* to penicillin is becoming more common, full penicillin resistance in these strains is still rare. Ceftriaxone and cefotaxime remain effective against virtually all strains (Chen et al., 2023). Chloramphenicol can be used as an alternative in the treatment of meningitis but is now less commonly used due to adverse effects (e.g., bone marrow aplasia) and the availability of safer options. Even though rifampicin and ciprofloxacin are used for post-exposure prophylaxis (chemoprophylaxis), resistance to them can also occur (Pickering, 2000; Albrecht, 2011; Berry et al., 2024). The overall resistance to most used antibiotics such as ceftriaxone, cefotaxime, ciprofloxacin, and rifampin is low, ranging from 1 to 3.4%. However, non-sensitivity to penicillin, as the first-line antibiotic against *N. meningitidis*, is higher (27.2%) (Albrecht, 2011).

*Streptococcus pneumoniae* is a Gram-positive bacterium that also colonizes the surface of the nasopharynx (carrier). It can cause milder respiratory infections, such as middle ear infections and sinusitis, as well as more severe diseases, including pneumonia (with or without sepsis) and meningitis. The introduction of pneumococcal conjugate vaccines in childhood immunization programs in many countries has led to a significant decrease in severe invasive pneumococcal diseases (IPD) among vaccinated children. However, infections caused by non-vaccine types have simultaneously increased, causing invasive pneumococcal diseases among unvaccinated individuals (such as older adults) (Narciso et al., 2025). Furthermore, *S. pneumoniae* as an etiological agent of meningitis causes serious complications much more frequently than other bacteria and may be responsible for recurrent meningitis (Skoczyńska and Hryniewicz, 2003; Albrecht, 2011). Moreover, meningitis mortality can reach 20-30% and rises to 60% in those with invasive meningococcal disease, which includes meningitis in addition to sepsis (Chen et al., 2021).

In the treatment of invasive pneumococcal infections (Invasive Pneumococcal Disease, IPD), broad-spectrum antibiotics are used. In empirical therapy for severe infections, it includes third-generation cephalosporins (cefotaxime, ceftriaxone). Particularly in meningitis, vancomycin (intravenously) is administered along with cefotaxime or ceftriaxone. If the strain is sensitive, penicillin G or ampicillin is administered (based on the antibiogram). In some cases, especially in adults, for example, in the treatment of severe pneumonia or in the case of β-lactam allergy, fluoroquinolones (moxifloxacin or levofloxacin) are used (Woodhead et al., 2011; Domínguez-Alegría et al., 2018).

A serious problem due to clinical and epidemiological consequences is the resistance of *S. pneumoniae* strains to penicillin, as these are usually multidrug-resistant strains that, in addition to resistance to β-lactams, may also be resistant to tetracyclines, macrolides, lincosamides, cotrimoxazole, and chloramphenicol, which significantly limits therapeutic options (Skoczyńska and Hryniewicz, 2003). The choice of empiric antibiotic treatment of meningitis is determined by the patient’s age and the regional rate of decreased susceptibility to penicillin and third-generation cephalosporins (**Table 2**). For example, in South Korea, over 80% of strains isolated in 2012–2019 were resistant to penicillin, while only 23,9% resistance was observed in Spain strains recovered in 2004-2020 (Kim et al., 2021; Sempere et al., 2022). What is worth mentioning is that the penicillin-resistant isolates (PRSP) should be examined for susceptibility to each cephalosporin used in the hospital. Such a procedure helps take into consideration the mosaic structure of genes responsible for this resistance, as it differently influences the activity of each β-lactam (Skoczyńska and Hryniewicz, 2003). In case of cephalosporin resistance certain combinations can be used: vancomycin plus rifampicin, vancomycin plus ceftriaxone or cefotaxime, or rifampicin plus ceftriaxone or cefotaxime. However, it’s still unclear if adding vancomycin or rifampicin to a third-generation cephalosporin is truly beneficial in pneumococcal meningitis, as their efficacy in cephalosporin-resistant pneumococci has only been tested on animals (Van De Beek et al., 2016).

**Table 2:** Regional rates of penicillin non-susceptibility in collected strains of *S. pneuomoniae* (CNS – central nervous system; IPD – invasive pneuomococcal disease).

Country/region	Years of strains collection	Type of infection	Penicillin resistant and non-susceptible strains in last year of analysis [%]	Other common resistances (<25% of strains)	Ref.
Spain	2004-2020	various	23,9	**- **amoxicillin **- **erythromycin **- **cefixime **- **cefpodoxime	(Sempere et al., 2022)
South Korea	2012-2019	CNS infection	85,7	**- **clindamycin **- **ceftriaxone **- **erythromycin **- **cotrimoxazole **- **tetracycline	(Kim et al., 2021)
Argentina	2006-2019	IPD	33,9	**- **erythromycin **- **tetracycline **- **doxycycline **- **cotrimoxazole	(Zintgraff et al., 2022)
Europe (8 countries)	2001-2003	various	24,6	**- **macrolides	(Reinert et al., 2005)
France	2009-2021	CNS infection	51,4	**- **amoxicillin **- **erythromycin	(Plainvert et al., 2023)
Madrid, Spain	2007-2021	IPD	31,6	no data	(De Miguel et al., 2023)

*Haemophilus influenzae* is a Gram-negative coccobacillus commonly colonizing the nasopharynx. In patients with disturbed systemic health, it mostly leads to airway mucosal infections. Some strains produce a capsule, whose antigenic differences serve as the basis for dividing this species into 6 serological types (a-f). The most dangerous infections are mainly caused by serotype b (Hib). This serotype is responsible for over 90% of meningitis caused by this species, as well as other invasive infections such as sepsis, pneumonia, epiglottitis, osteomyelitis, arthritis, and subcutaneous tissue infections (Moxon and Murphy, 2000). It is crucial to mention that in the past years the incidence of Hib infections decreased due to the worldwide availability of a conjugate vaccination against this species. Nevertheless, when such an infection occurs, it is usually treated with beta-lactams, with aminopenicillins and cephalosporins being the first choice.

From a clinical point of view, the most important resistance mechanism occurring in *H. influenzae* is resistance to β-lactam antibiotics associated with the production of β-lactamase and/or changes in penicillin-binding proteins, PBP3. Due to resistance to this group of drugs, *Haemophilus* bacilli can be divided into 3 groups: β-lactamase-positive strains, resistant to ampicillin (*BLPAR*); β-lactamase-negative, ampicillin-resistant strains (*BLNAR*); and β-lactamase-positive strains, resistant to the combination of amoxicillin and clavulanic acid (*BLPACR)*. In a meta-analysis published in 2023, covering 19,787 isolates from 2003–2023, it was found that approximately 34.9% of *H. influenzae* isolates produce β-lactamase, and about 23.1% are multidrug-resistant (MDR) strains (Abavisani et al., 2024). Other antibiotics used in therapy are fluoroquinolones, macrolides, chloramphenicol, and tetracyclines. However, for all of them, resistance mechanisms were also detected, implying possible treatment concerns in the future (Wen et al., 2020).

*Klebsiella pneumoniae* is a Gram-negative pathogen that mostly causes primary pneumonia and urinary tract infections, especially in those with weakened immune systems (Li et al., 2014). That is why it is often responsible for healthcare-associated infections (HAI). Antibiotics such as carbapenems, cephalosporins, fluoroquinolones, and aminoglycosides are commonly used to treat *K. pneumoniae* infections. However, *K. pneumoniae* has multiple mechanisms that make it resistant to various classes of antibiotics (Moya and Maicas, 2020). These are the production of β-lactamases, ESBLs, carbapenemases, changes in outer membrane permeability, efflux pumps, and mutations in target genes (Moya and Maicas, 2020; Kot et al., 2025). A major threat is the multidrug-resistant and MDR-hv strains, which exhibit resistance to distinct classes of antibiotics, including beta-lactams, aminoglycosides, quinolones, tigecycline, and polymyxins [19]. According to the ECDC−WHO report “Surveillance of antimicrobial resistance in Europe, 2023,” resistance to third-generation cephalosporins in many countries of southern and eastern Europe exceeds 50% (ECDC-WHO, 2024). Moreover, a recent meta-analysis of 77 studies from 17 countries revealed high resistance rates observed in hypervirulent strains (hvKp) against various antibiotics like ampicillin-sulbactam (45.3%), cefazolin (38.1%), cefuroxime (26.7%), cefotaxime (65,8%), and ceftazidime (57,1%), as well as the fourth-generation cephalosporin cefepime (51.3%). The last resort carbapenems - imipenem (45.7%), meropenem (51.0%), and ertapenem (40.6%) - also revealed substantial resistance (Russo and Marr, 2019; Riwu et al., 2022; Beig et al., 2024a). On top of that, heteroresistance of *K. pneumoniae* strains was reported in recent years - a phenomenon where subpopulations of the seemingly susceptible bacteria exhibit resistance. This process has been associated with increased morbidity and mortality rates and can involve multiple mechanisms of resistance (Lin et al., 2024).

*Listeria monocytogenes* is a Gram-positive foodborne pathogen, mostly transferred through contaminated dairy and meat products. This bacterium is responsible for listeriosis - an infection that can manifest as bacteremia, meningoencephalitis, or fetal-placental infection in pregnant women (Matle et al., 2020; Disson et al., 2021). *L. monocytogenes* can cause meningitis in people with weakened immune systems (often after kidney transplants, in patients undergoing corticosteroid therapy, alcoholics, and patients with cancers, diabetes, liver diseases, and chronic kidney diseases). Although illnesses caused by this microorganism are not among the most common, the mortality rate among patients is very high, reaching even 20-70% (Ramaswamy et al., 2007; Yau et al., 2018). *L. monocytogenes* standard therapy is based on amoxicillin or ampicillin, often combined with gentamicin (Baquero et al., 2020). Aminopenicillins can be replaced by cotrimoxazole, fluoroquinolones, rifampicin, or linezolid. Vancomycin can also be occasionally used in nonmeningeal infections, while erythromycin is used for listeriosis during pregnancy. Most isolates from clinical as well as food-borne and environmental sources are generally susceptible to a wide range of antibiotics, but occasionally resistance to tetracycline, vancomycin, and ampicillin has been observed (Depardieu et al., 2007; Olaimat et al., 2018; Yan et al., 2019; Baquero et al., 2020).

*L. monocytogenes* most often enters the body through contaminated food: unpasteurized milk and its products, soft mold cheeses (camembert, brie), cold cuts and pâtés, as well as smoked fish (Jordan et al., 2018). *Listeria monocytogenes* is quite resistant to harsh environmental conditions, including low temperatures (it can grow in the refrigerator), high salt concentrations, or low pH. It is particularly dangerous in food that is stored for long periods or improperly processed (EFSA Panel on Biological Hazards (BIOHAZ) et al., 2018). Therefore, in the fight against this bacterium in food production environments/processing facilities, great emphasis is placed on monitoring methods, disinfection, and biocontrol based on bacteriophages or endolysins (Norton et al., 2001; Gray et al., 2018).

*Staphylococcus aureus* is a Gram-positive pathogen commonly causing various types of infections, e.g., post-surgery skin infections. It may also cause more severe and invasive conditions like pneumonia, osteomyelitis, arthritis, cardiovascular infections, and meningitis – rare, but very severe when they occur (Cheung et al., 2021). The treatment of infections caused by *Staphylococcus aureus* depends on the strain (methicillin-resistant – MRSA – or sensitive – MSSA), the site of infection, the clinical severity, the patient’s condition, and the results of the antibiotic susceptibility testing. In the case of skin, soft tissue and joint infections and endocarditis, cloxacillin or flucloxacillin are used, and in the case of penicillin allergy, cefazolin, clindamycin, doxycycline, or oxazolidinones are used. In severe infections (e.g., sepsis, lungs, bones, endocardium) caused by MRSA strains, vancomycin, linezolid, daptomycin, or ceftaroline are used, with vancomycin as the first-line drug. In infections involving biofilm (catheter or implant infections), combination therapy is used, mainly with the addition of rifampicin or fosfomycin (Liu et al., 2011).

Problems with drug resistance in *Staphylococcus aureus* are a serious clinical and epidemiological challenge — both in hospitals and in outpatient care. The overuse of penicillin in the treatment of *S. aureus* infections resulted in a high prevalence of penicillin resistance among the pathogenic strains. Penicillin-resistant *S. aureus* can produce penicillinase, which hydrolyzes the penicillin β-lactam ring. For this reason, penicillin was exchanged for methicillin in *S. aureus* treatment, as it is resistant to penicillinases. Methicillin was effective for a few years, after which methicillin-resistant *Staphylococcus aureus* (MRSA) was subsequently isolated. Vancomycin is usually considered to be the drug of choice for MRSA, but unfortunately even for this drug the resistance arose in time (VISA and VRSA strains) (Guo et al., 2020; Marciniak et al., 2024). When methicillin and vancomycin resistances coexist, the last line of defense is administration of daptomycin or linezolid. Unfortunately, *S. aureus* is also able to develop resistance to both of those antibiotics, making such infections extremely hard to treat (Zhou et al., 2025).

The percentage of invasive *S. aureus* isolates resistant to methicillin (MRSA) in EU countries is approximately 15.8%, and the number of MRSA cases isolated from bloodstream infections is 4.64 per 100,000 in 2023 (ECDC, 2023). The problem with *S. aureus* strains is that MRSA is resistant to many antibiotics, and treatment relies on expensive or more toxic drugs (vancomycin, linezolid, and daptomycin). In addition, MRSA can colonize catheters, implants, and wounds, where it forms a biofilm that increases strain resistance and complicates treatment. It should be noted that MRSA causes not only skin infections but also invasive infections, including sepsis, in which mortality caused by MRSA strains can reach 30–40% (Li et al., 2025).

While most cases of meningitis in adults are caused by the aforementioned pathogens, in some cases less common bacteria may be responsible. Antibiotic therapy in infrequent etiology infections varies strongly not only in the class of antibiotics used but also in the way of their administration. Moreover, these rare agents also often show resistance to numerous antimicrobials, implying the need for another, more effective treatment (**Table 3**). For example, treatment of *M. tuberculosis* infections usually engage the use of different antibiotics like rifampin, isoniazid, and pyrazinamide simultaneously. That’s why it’s extremely important to explore other ways of fighting these bacteria, such as, e.g., phage therapy. This may not only help decrease the risk of meningitis and its sequelae but also prepare us to fight against meningitis caused by the MDR strains.

**Table 3:** General antibiotic therapy of less common meningitidis-associated pathogens.

Species and their most common disease	Most common in primary infection		Meningitis	Ref.
	First line antibiotics	Possible acquired resistance		
Mycobacterium tuberculosis (Tuberculosis)	▪ rifampicin ▪ isoniazid ▪ rifapentine ▪ moxifloxacin ▪ pyrazinamide	▪ rifampicin ▪ multidrug	▪ rifampin ▪ isoniazid ▪ ethambutol ▪ pyrazinamide(supplementary fluoroquinolones)	(Ye et al., 2021; Lin, 2024; WHO, 2025)
Cutibacterium (Acne)	▪ clindamycin ▪ doxycycline	▪ erythromycin ▪ clindamycin	▪ penicillin G ▪ ceftriaxone	(Tunkel et al., 2017; Rimon et al., 2023; National Institute for Health and Care Excellence; Beig et al., 2024b)
Leptospira (Leptospirosis)	▪ doxycycline ▪ penicillin	▪ β-lactams	▪ doxycycline ▪ ampicillin ▪ ceftriaxone	(Rajapakse, 2022; Pineda et al., 2024; Mendu et al., 2025)
Brucella (Brucellosis)	▪ doxycycline ▪ streptomycin	▪ noncommon	▪ doxycycline ▪ rifampicin ▪ ceftriaxone ▪ trimethoprim-sulfamethoxazole	(Erdem et al., 2012; Qureshi et al., 2023)
Bacteroides fragilis	▪ carbapenems ▪ tazobactam/ piperacillin ▪ metronidazole	▪ carbapenems ▪ tazobactam/ piperacillin	▪ metronidazole	(Jasemi et al., 2021; Yekani et al., 2022; Kato et al., 2025)
Fusobacterium necrophorum	▪ Metronidazole + β-lactam antibiotics	▪ β-lactam	▪ amoxicillin /clavulanic acid ▪ clindamycin ▪ metronidazole	(Garimella et al., 2004; Lee et al., 2020)
Clostridium perfringens	▪ penicillin G ▪ ampicillin ▪ clindamycin ▪ metronidazole	▪ β-lactams	▪ ampicillin ▪ clindamycin ▪ metronidazole	(Fourie et al., 2020)

## 4 Bacteriophages against meningitis pathogens

**Figure 2:**
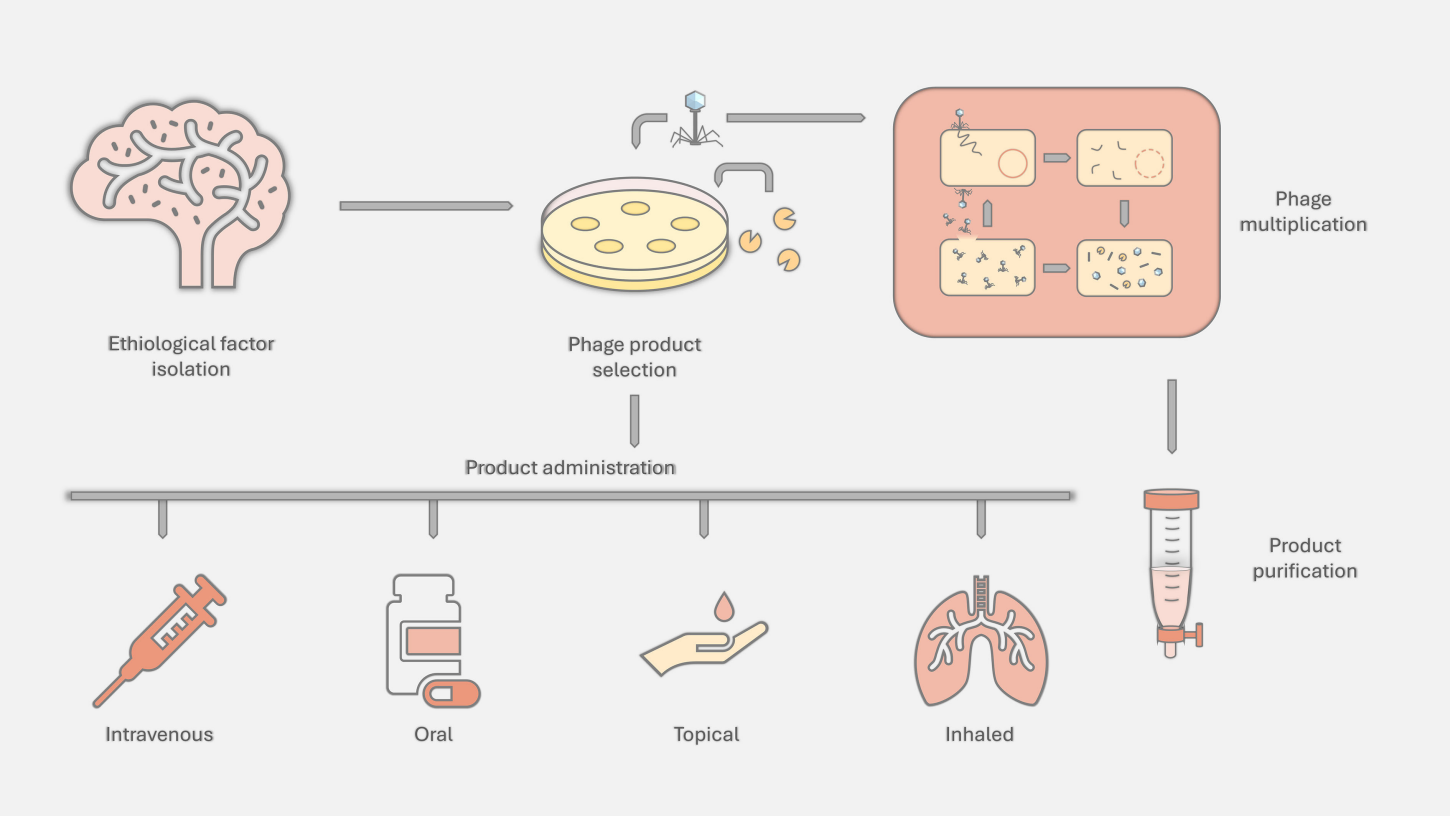
Scheme of characterized bacteriophages/enzymes preparation for phage therapy and their most common administration routes.

Bacteriophage therapy treats bacterial infections by using bacteriophages - viruses that specifically infect and lyse bacteria. Phages eliminate the pathogenic cells without harming human tissues or beneficial microbiota. In addition, many phage derivatives, like depolymerases, are currently being investigated as antibacterial compounds (Easwaran et al., 2025; Faccin et al., 2025). Compared to antibiotics, these entities are easy to discover, have a minimal influence on natural flora, are often effective with a single-dose, and can also reduce the virulence and resistance of the pathogen (Guo et al., 2024). However, there are only a few phage-based drugs allowed to be used in human infections (usually in the form of experimental therapy) - most probably due to concerns like the risk of toxin gene possession (Yang et al., 2023). Bacteriophages can undergo two primary life cycles: the lytic cycle, characterized by rapid replication within the host bacterium leading to its lysis, and the lysogenic cycle, in which the phage genome integrates into the bacterial chromosome and remains dormant until triggered to enter the lytic phase. In phage therapy, the lytic cycle is preferred, as it leads to an immediate destruction of a bacterial cell, ensuring rapid reduction of the bacterial load without the risk of transferring harmful genes (e.g., antibiotic resistance genes), which can occur in the lysogenic cycle. Therefore, it is important to precisely characterize both the activity of the phage and its genome (Hibstu et al., 2022). Another concern is the development of phage resistance. This phenomenon occurs frequently but can be simply combated by using phage cocktails, combinations with antibiotics, or phage-encoded enzymes (Chen et al., 2022, 2024; Kunisch et al., 2024). When the product is already defined, it is tested for activity toward pathogen isolates and then directly applied to the patient. However, in some cases phages are then multiplied to produce the amount of particles/enzymes needed for therapy, often using the isolated pathogenic strain (**Figure 2**).

In the case of pathogens mentioned above, their phages are broadly isolated and evaluated, except for viruses against *N. meningitidis* due to the high effectiveness of antibiotic therapy and low risk of resistance. Even though most of the experiments were performed using cell cultures or studies using mouse models and, in some cases, were aimed against primary infections, the promising results still imply the importance of further bacteriophage research. Additionally, clinical studies on phage therapy may lead to the development of satisfactory alternatives to antibiotics in the treatment of both primary and more complex infections, such as meningitis.

### 4.1 Streptococcus pneumoniae

Due to the rapid variability of *Streptococcus pneumoniae* strains and serotypes, it is difficult to develop a “universal” phage that would lyse most strains. Moreover, there is a limited amount of data available in the literature that well characterizes phages against *S. pneumoniae*. Therefore, research on phage therapy primarily focuses on phage endolysins – enzymes that degrade the bacterial cell wall. Unlike antibiotics, lysin can also destroy the cell walls of non-growing bacteria. Additionally, lysin is highly specific to bacterial species, as well as to the phages that produce it (Starkevič et al., 2015). So far, several phage lysins acting against *S. pneumonia*e strains have been investigated (Cpl-1, Cpl-7/Cpl-7S, Cpl-711, ClyJ, SP-CHAP, and 23TH_48) (Loeffler et al., 2001; Grandgirard et al., 2008; Doehn et al., 2013; Díez-Martínez et al., 2013, 2015; Corsini et al., 2018; Yang et al., 2019; Van Der Kamp et al., 2020; Silva et al., 2021; Alreja et al., 2024; Vander Elst et al., 2025).

Lysin Cpl-1 is an enzyme with hydrolase activity produced by some of the *S. pneumoniae* phages, which is capable of degrading peptidoglycan – the main component of the bacterial cell wall (Grandgirard et al., 2008). In the studies by Grandgirard et al (Grandgirard et al., 2008), recombinant Cpl-1, specific for *S. pneumoniae*, was evaluated for antimicrobial treatment in experimental pneumococcal meningitis using Wistar rats. Lysin Cpl-1 showed the ability to kill all of the tested serotypes of *S. pneumoniae in vitro*. It was also demonstrated that Cpl-1 has an ability to kill *S. pneumoniae* in an infant rat model of bacterial meningitis when injected intracisternally or intraperitoneally. An intracisternal injection of lysin Cpl-1 decreased the CSF bacterial counts to below the detection limit 30 min after the injection. This effect lasted up to 3 h, and after that bacteria were detected again. An intraperitoneal injection resulted in a constant Cpl-1 level in plasma and CSF for a longer period of time after the injection, which might suggest that this route of administration is more adequate. In summary, it was concluded that the Cpl-1 lysin may be promising as an alternative treatment option for pneumococcal meningitis (Grandgirard et al., 2008). Doehn et al (Doehn et al., 2013). tested the effect of a single dose of recombinant, aerosolized Cpl-1 (0.4 mg) on mice with severe pneumonia caused by pneumococci and observed its effect through 10 days. Endolysin effectively reduced the number of bacteria in the lungs and prevented bacteremia. Although the levels of inflammatory cytokines increased shortly after Cpl-1 inhalation, the mice quickly recovered, as evidenced by weight gain, and the inflammatory infiltrates in the lungs subsided, leading to an 80% reduction in mortality. Díez-Martínez et al (Díez-Martínez et al., 2015). obtained new chimeric phage endolysins (Cp1-711), with enhanced bactericidal activity, by exchanging structural components of two pneumococcal phage enzymes: Cpl-1 (the best lysin tested so far) and Cpl-7S. The bactericidal activity of the new chimeric lysins was tested on bacteria by adding purified enzymes at various concentrations to bacterial suspensions, and living cells were measured after 1 hour. The bactericidal ability of Cpl-711 was tested in a mouse bacteremia model after the survival of mice following injection of various amounts of the enzyme (25–500 μg). The ability of Cpl-711 to limit pneumococcal biofilm formation was also examined. The chimera Cpl-711 significantly enhanced the killing activity of the parent phage lysins, Cpl-1 and Cpl-7S, against pneumococcal bacteria, including multi-resistant strains. Specifically, Cpl-711 at a concentration of 5 μg/ml killed ≥7.5 log of the R6 pneumococcal strain. Cpl-711 also reduced pneumococcal biofilm formation and killed 4 logs of the bacterial population at a concentration of 1 μg/ml. Mice infected intraperitoneally with the pneumococcal strain D39_IU were protected after a single intraperitoneal injection of Cpl-711 one hour later, providing about 50% greater protection than with Cpl-1, which action resulted in a lower number of mice rescued from the lethal infection. This enzyme is a perfect example of a promising therapeutic prospect for the treatment of multi-drug-resistant pneumococcal infections, and swapping domains between phage lysins allows the construction of new chimeric enzymes with high bactericidal activity and different substrate ranges (Díez-Martínez et al., 2013). Alreja et al (Alreja et al., 2024). presented an endolysin targeted against *Streptococcus pneumoniae*, which also shows greater activity than Cpl-1, the best-characterized pneumococcal endolysin. Cysteine and histidine-dependent amidohydrolase/peptidase (CHAP) domains are widely represented in bacteriophage endolysins but have never been described in pneumococcal endolysins. SP-CHAP exhibited antimicrobial activity against all tested *S. pneumoniae* serotypes, including encapsulated and non-encapsulated pneumococci, and proved to be more active against 3 of 5 strains than the most studied pneumococcal endolysin. A colony-forming unit (CFU) assay showed a dose-dependent lytic activity, with a 7 log_10_ decrease in bacterial load after treatment with the highest concentration (100 µg/mL). Surprisingly, the Cpl-1 was able only to cause a 2 log drop. Its efficacy in combating pneumococcal biofilms *in vitro* and in a mouse nasopharynx colonization model was also demonstrated. In both models, the enzyme caused a greater reduction of bacterial count than when treated with Cpl-1. These findings highlight the therapeutic potential of SP-CHAP as an alternative to antibiotics in the treatment of *S. pneumoniae* infections. Van der Kamp et al (Van Der Kamp et al., 2020). identified two new Streptococcal phages from the oral microbiome, 23TH and SA01. Their lysins, 23TH_48 and SA01_53, were recombinantly produced, characterized, and tested for their activity against *S. pneumoniae* strains. They demonstrated that 23TH_48 has broader lytic activity beyond the *S. infantis* strain, with several *S. pneumoniae* isolates being highly sensitive to its lytic activity. Given this activity, 23TH_48 may prove to be a promising candidate for helping to combat pneumococcal infections. However, none of these new enzymes were tested *in vitro* or in clinical conditions. The possibilities of treating *S. pneumoniae* infections were also studied in so-called combination therapy, in which synergy between antibiotics and phage lysins was observed. For example, earlier mentioned lysin Cpl-1 is a promising addition to the standard antibacterial therapy because of its synergistic activity with penicillin, gentamicin, and Pal/LytA (other phage lytic enzymes) (Rodriguez-Cerrato et al., 2007; Grandgirard et al., 2008; Bustamante et al., 2010; Corsini et al., 2018). Systemic administration of Cpl-1 in combination with penicillin in mice infected with penicillin-resistant pneumococci effectively reduced bacteremia, and thanks to efficient penetration through the blood-brain barrier, it eliminated the bacterial load in the brain, resulting in an increase in survival (89%) with an asymptomatic course of infection. These findings strongly suggest that Cpl-1 may enhance antibiotic sensitivity in β-lactam- and macrolide-resistant *S. pneumoniae*, serving as a valuable complement to standard antibiotic therapy in cases of multidrug-resistant invasive pneumococcal disease (Vander Elst et al., 2025). Research has also been conducted on improving the stability and transport of lysins to *S. pneumoniae*. Silva et al (Silva et al., 2021). encapsulated the MSlys endolysin in deformable liposomes for targeted lysin delivery in the treatment of pneumococcal otitis media. Preclinical studies have shown that phage derivatives can effectively reduce *S. pneumoniae* colonies in *in vitro* and *in vivo* models and act synergistically with certain antibiotics (Djurkovic et al., 2005; Vouillamoz et al., 2013; Vander Elst et al., 2025).

### 4.2 Haemophilus influenzae

Gatea et al (Cheung et al., 2021). conducted a study in which they tested phage preparations against several bacteria, including *H. influenzae*, in a mouse HIF-1 bacterial sepsis model. Researchers demonstrated four bacteriophages extracted from sewage water with different activity against *H. influenzae* strains from their environment and other isolates (Gatea Kaabi and Musafer, 2020). Additionally, mixing the examined four phages increased the effectiveness of phage therapy to 100%, which is why the phage cocktail was then successfully used in cases of neonatal sepsis.

Adamczyk-Popławska et al (Adamczyk-Popławska et al., 2020). described a lytic gene set (holin endolysin) of phage HP1, which may be useful as a component of phage therapy or as an enzymatic component destroying *H. influenzae* bacteria. The activity of this phage may be attributed to the presence of *lys* and *hol* genes, products of which are accountable for the signal-arrest-release (SAR) mechanism of endolysin and pinholin, respectively (Starkevič et al., 2015). Researchers also discovered that pinholin has regulatory power over the activity of SAR-endolysin and thus plays a crucial role in the mechanism of cell lysis. That discovery supports the claim that for the best therapeutic outcome, a combination of both of those enzymes should be used. However, all tests were performed *in vitro*, implying the need for enzyme examinations in more models.

Interestingly, there are vaccines based on phages and their phagemids. In a brought-up case, phagemid **[**pBSKS::Φ6fm(Hin)**]** particles incorporated in *H. influenzae* were used as a mediator for a vaccination, later used to immunize rabbits. *H. influenzae* Rd30 cells release phagemids that contain their proteins, leading to their appearance and production of IgG antibodies that recognize both the host’s and the phages’ proteins. As it is said, phage NgoΦ6 can form phagemid particles from any Gram-negative bacteria, leading to immunization for that bacteria (Piekarowicz et al., 2022).

### 4.3 Klebsiella pneumoniae

Bacteriophages against *Klebsiella pneumoniae* strains are an area of rapidly developing research, particularly in the context of the increasing antibiotic resistance of this pathogen. The effectiveness of phage therapy against diseases such as wounds and soft tissue infections, bacteremia, and pneumonia was studied in mouse models that accurately reflect the range of diseases caused by these bacteria in humans (Vinodkumar et al., 2005; Kumari et al., 2010; Cao et al., 2022). Kumari and colleagues evaluated the therapeutic potential of five *K. pneumoniae* phages isolated from sewage material (Kumari et al., 2010). One of the phages, Kpn5, showed the highest lytic activity *in vitro* and was tested in burn wound BALB/c mouse models (Kumari, 2009; Kumari et al., 2010). It was found that a single intravenous dose of phages at MOI = 1, immediately after bacteria application, was able to save 96.66% of infected mice. However, the delays (6, 12, 18, and 24 h) in the administration of phage resulted in the lower survival rate, with only the 24 h delay resulting in no survivals. Despite that, the observed protection effect in other delays stabilized on day 4, and there were no changes in mortality to the end of the experiment (day 20).

The combination of antibacterial drugs with phages and phage cocktails is a novel approach to reducing bacterial resistance. Many researchers use this new approach in treating phage-resistant *K. pneumoniae* strains (Verma et al., 2009; Gu et al., 2012; Chadha et al., 2016). Chadha et al (Chadha et al., 2016). observed greater efficacy of the phage cocktail, containing phages Kpn1, Kpn2, Kpn3, Kpn4, and Kpn5, than single phages in treating burn wound infection in BALB/c mice. The reduction of bacterial load occurred in a shorter time compared to single phages, and mice from the cocktail-treated group had the highest wound contraction (~ 91.66%) at the end of the experiment (day 20). Verma and colleagues (Verma et al., 2009) applied a combination of ciprofloxacin and lytic phages in the treatment of *K. pneumoniae* biofilms. The authors found that this combination reduces the development of *K. pneumoniae* strains resistant to ciprofloxacin and phage-resistant strains and has greater anti-biofilm activity than monotherapy. A phage cocktail (a combination of three lytic phages GH-K1, GH-K2, and GH-K3), which have different but overlapping host specificities, was used in the treatment of bacteremia caused by the *K. pneumoniae* K7 strain in a mouse model. The results obtained for phages injected intraperitoneally 30 min after the K7 challenge suggest that the phage cocktail reduced the production of phage-resistant K7 variants. Moreover, the phage cocktail administered to mice with bacteremia increased mouse health and survival rates compared to the control or phages alone (Gu et al., 2012). In the cocktail-treated group, only 1 of 5 mice developed a severe infection, while for the phages alone, 14 of 15 mice developed a severe or moribund state. Due to the possible hematogenous seeding to CSF, such experiments are the second-best studies (after direct meningitis studies) that can be used to estimate the phages’ viability in treating *K. pneumoniae* meningitis. Thus, often representing the promising results in bacteria eradication and saving the lives of even 100% of infected mice (**Table 4**).

**Table 4:** Overview of mice studies on phage therapy for *Klebsiella pneumoniae* bacteremia (PFU – plaque forming unit; CFU – colony forming unit).

Study	Phage	Clearence of bacteria in blood	Endpoints and limitations
(Gu et al., 2012)	GH-K1 (3.0×10^7^ PFU/mouse) GH-K2 (3.0×10^5^ PFU/mouse) GH-K3 (3.0×10^6^ PFU/mouse) Phage cocktail (3.0×10^4^ PFU/mouse)	*Injected intraperitoneally 30 mins after infection:* GH-K1 - 3.6×10^6^ CFU/mL → 3 ×10^2^ CFU/mL in 2 h GH-K2 - 3.6×10^6^ CFU/mL → 2 ×10^2^ CFU/mL in 2 h GH-K3 - 3.6×10^6^ CFU/mL → 1.1×10^3^ CFU/mL in 2 h Phage cocktail - 3.6×10^6^ CFU/mL → 3.6×10^1^ CFU/mL in 2 h	30 days observation: ▪No adverse effects detected ▪For each phage/cocktail a titer with 100% survival rate was determined
(Hesse et al., 2021)	P1 [Pharr] (5 × 10^7^ PFU/mouse) P2 [ϕKpNIH-2] (5 × 10^7^ PFU/mouse) Phage cocktail (2.5 × 10^7^ PFU of each phage)	*Injected intraperitoneally 1 h after infection:* P1–10^6^ CFU/mL → ~ 10^3^ CFU/mL after 24 h P2–10^6^ CFU/mL → ~ 10^3^ CFU/mL after 24 h Phage cocktail - 10^6^ CFU/mL → ~ 10^3^ CFU/mL after 24 h	10 days observation: ▪Survival rates - 93%, 80%, 100% (P1, P2, cocktail) ▪Appearance of phage resistant mutants with less ratio when cocktail was used
(Shi et al., 2021)	Kpssk3 (10^8^ PFU/mouse)	*Injected intraperitoneally 3 h after infection:* no data	7 days observation: ▪Survival rate – 100% ▪Slight changes of gut microbiota ▪Appearance of phage resistant mutants
(Asghar et al., 2022)	A¥L (10^8^ PFU/ml) A¥M (10^8^ PFU/ml)	*Injected intraperitoneally 1 h after infection:* A¥L (in 120 h) - 9.68 ± 0.44 log10 CFU/ml → 3.125 ± 0.49 log10 CFU/ml A¥M (in 120 h) - 9.68 ± 0.44 log10 CFU/ml → 4.806 ± 0.44 log10 CFU/ml Phage cocktail (in 120 h) - 9.68 ± 0.44 log10 CFU/ml → 2.95 ± 0.33 log10 CFU/ml	7 days observation: ▪Survival rate – 100% (A¥L and cocktail), 83% (A¥M) ▪no data about adverse effects
(Singh et al., 2022)	Phage cocktail [ΦKpBHU4, ΦKpBHU7, ΦKpBHU14] (10^5^ PFU/mouse)	*Injected intraperitoneally:* ▪simultaneously with infection: - below detection level in 24 hours (from 8 × 10^7^ CFU/ml) ▪6 hours after infection and with single doses for six days: - complete eradication in 6 days (8 × 10^7^)	6 days observation: ▪Survival rate – 100% ▪High endotoxin release in mice treated with 10^12^ PFU/ml
(Liang et al., 2023)	BL02 (10^8^ PFU/mouse)	*Injected intraperitoneally 1 h after infection:* no data	7 days observation: ▪Survival rate – 71.43% ▪Higher effectivity then antibiotics (tigecycline – 28.57% or ceftazidime/avibactam – 42.86%) ▪no data about adverse effects
(Tang et al., 2024)	φFK1979 (10^3–^10^12^ PFU/mL)	*Injected intraperitoneally 2 and 18 h after infection:* no data	7 days observation: ▪Survival rates – 100% in immunocompetent mice (10^6–^10^12^ PFU/mL), 0% (10^3^ PFU/mL) ▪For immunodeficient mice efficacy increased by addition of other phage

The use of peptides derived from phages instead of the whole phage represents another alternative therapy option for multidrug-resistant *K. pneumoniae*. A polysaccharide depolymerase derived from KP36, named depoKP36, was identified, cloned, and expressed in *E. coli* (Majkowska-Skrobek et al., 2016). This enzyme produced a halo in a spot test on a bacterial biofilm on an agar plate. Furthermore, the efficacy of this enzyme was evaluated in the treatment of infections in *G. mellonella*. All larvae died without treatment, whereas following post-infection enzyme treatment, up to 40% of the larvae survived.

Although currently there are no studies using the aforementioned phages to treat specifically meningitis, there are various cases where the combination of antibiotics and phages/phage cocktails was used to treat *K. pneumoniae*. Most of them bring forward rather promising results—the combination of these two types of antimicrobials leads to a total eradication of *K. pneumoniae* or resolution of symptoms. A Dutch clinician successfully treated a kidney transplant patient with recurrent urinary tract infection caused by ESBL-producing K. *pneumoniae* bacteria using a combination of phages and meropenem (González-Menéndez et al., 2018). Treatment with meropenem alone failed. The patient received phage therapy from the Eliava Institute (Georgia) and remained infection-free after the therapy. This outcome demonstrated that the combination therapy was effective (Kuipers et al., 2019). Italian clinicians also effectively used a phage cocktail in the treatment of invasive MDR *K. pneumoniae* infections containing KPC-3 (ST307). At the same time, phages were applied for the decolonization of bacteria from the intestines, which is a key step in preventing future infections and the development of resistant bacteria. The results showed that this treatment has no adverse effects (Corbellino et al., 2020).

### 4.4 Listeria monocytogenes

Although bacteriophages are not currently used in the treatment of human *L. monocytogenes* infections due to the intracellular nature of the bacilli, they are used as an alternative or supplement to conventional disinfection methods, both in healthcare and the food industry, to prevent foodborne listeriosis (Palma and Qi, 2024). Products at risk include, among other things, pasteurized whole milk, cheeses, ice creams, fruits, vegetables, and various (both raw and ready-to-eat) meat and fish products (Schlech, 2019). Phage-based solutions, such as Listex™ and ListShield™, have already acquired GRAS (Generally Recognized as Safe) status and are widely used in the USA.

Studies on the preparation ListShield™, a commercially available bacteriophage cocktail that specifically targets *Listeria monocytogenes*, showed a reduction of *L. monocytogenes* presence in lettuce, cheese, apples, and smoked salmon, as well as in frozen ready-to-eat meals. Additionally, the ListShield™ preparation removed *L. monocytogenes* from naturally contaminated smoked salmon while not altering the organoleptic properties of the ready-to-eat meats (Perera et al., 2015). ListShield™ showed promising results, with a study done on Spanish dry-cured ham suggesting reduction below the detection limit (10 CFU/cm²) for lower contamination levels (10³ CFU/cm² and 10^4^ CFU/cm²) and reduction by 3.5 log units for the higher contamination level (10^5^ CFU/cm²), both after storage at 4 °C for 14 days (Grigore-Gurgu et al., 2024).

The preparation Listex, on the other hand, contains the bacteriophage P100, which was originally isolated from wastewater from a dairy plant and selected based on its ability to lyse *L. monocytogenes*. It belongs to the family Myoviridae, subfamily Spounavirinae, and genus Twortlikevirus and has a DNA genome of 131 kbp with 174 open reading frames (ORFs) encoding proteins (GenBank reference: DQ004855) (Carlton et al., 2005; Klumpp and Loessner, 2013). Phage P100 was developed as a product that obtained GRAS status granted by the FDA/USDA for use in all food materials. This phage was also used in numerous studies demonstrating its effectiveness in removing *Listeria* contaminants from fish, eliminating Listeria biofilms from stainless steel surfaces, and preventing the presence of *Listeria* in cooked ham (Holck and Berg, 2009; Soni and Nannapaneni, 2010b, 2010a; Grigore-Gurgu et al., 2024). In 2016 the EFSA Panel on Biological Hazard released an evaluation of the safety and efficacy of Listex™ (EFSA Panel on Biological Hazards (BIOHAZ), 2016). EFSA concluded that bacteriophage P100 doesn’t pose a toxicological risk, as it’s strictly lytic to bacteria and unable to transduce bacterial DNA. Moreover, although not many studies were conducted on naturally contaminated food (only 1 of the 33 considered during the evaluation), Listex™ proved mostly effective on the artificially inoculated ready-to-eat (RTE) samples (in 10^9 PFU/cm² concentration). It’s worth noting that EFSA clearly advises for Listex™ to be used as an additional tool to GHP and GMP, not as a standalone solution, as the product does not fully eliminate *L. monocytogenes* from samples with concentration levels of 100-10,000 CFU/g. Even though the conclusions of the evaluation seem promising, EFSA stated that more studies on naturally contaminated RTE foods should be undertaken before the product is authorized and used. Additionally, it is underlined that if the product were to be authorized and used, FBO should have additional means to control its dose and *L. monocytogenes* susceptibility to P100.

Biocontrol of *L. monocytogenes* using lytic bacteriophage preparations, such as ListShield™ or Listex, may offer an environmentally friendly, green approach to reducing the risk of listeriosis associated with the consumption of various food products that may be contaminated with *L. monocytogenes*. Although there were attempts to bring Listex™ to the EU market, the efforts have reached a standstill due to current regulations. Despite EFSA’s 2016 review, which shed a cautiously positive light on Listex™’s future in the European Union, the Standing Committee on Plants, Animals, Food and Feed did not pursue the approval process for Listex™, neither in the “decontaminant” nor in the “non-decontaminating processing aid” category (EFSA Panel on Biological Hazards (BIOHAZ), 2016). The convoluted legal route for now ended with a preliminary ruling (22 February 2024) that stated that Listex™ P100 still requires the European Commission’s approval for it to be distributed and used in the member states.

Phages can also be used to disinfect surfaces and equipment in food processing facilities in situations where conventional agents are ineffective or toxic. Their selectivity means they do not destroy biofilms indiscriminately—only those formed by *Listeria.* In a study conducted by Reinhard et al., various surfaces (stainless steel, thermoplastic conveyor belts, and epoxy floors) were tested for *L. innocua* reduction using phage P100 under different conditions: temperature (4 °C and 20 °C), phage concentration (1% and 5%), and exposure time (1 hour and 3 hours) (Reinhard et al., 2020). Listeria reduction was observed on all three materials under all tested conditions.

### 4.5 Staphylococcus aureus

Most research on phage therapy for staphylococcal infections is based on animal models or *in vitro* studies, while the number of well-documented clinical studies in humans is still limited. Bacteriophages against *S. aureus* can be found in nature, for example, in nasal swabs, soil, sheep feces, and even in cows with mastitis (Teng et al., 2022). They can be administered individually or in cocktails, such as the StaphLyse™ comprising SAML-4, SAML-12, SAML-150, SAML-229, and SATA-8505 phages (Brouillette et al., 2023). The difference between the efficacy of individual phages and phage cocktails is clearly visible among many studies. For example, one study showed that single phage samples (APTC-SA-2, APTC-SA-4, APTC-SA-12, or APTC-SA-13) are effective against 80%-95% of examined *S. aureus* isolates, while a phage cocktail (APTC-C-SA01, composition as above) was active against over 98% (Liu et al., 2022). Aside from that, the conjunction of phage therapy and antibiotic therapy shows their synergism - which, in the case of, e.g., phage Sb-1 and oxacillin, increases its effectiveness to 90% from 35% of Sb-1 phage alone (Simon et al., 2021). This synergistic effect was first discovered when sublethal portions of antibiotics led to higher production of lytic bacteriophages. In addition, phages lower bacteria’s MIC, increasing their susceptibility to antibiotics. Moreover, in the mouse model using *S. aureus* phages, TNF-α and interleukin-6 levels were more notably reduced than in the ceftiofur sodium therapy model, which points to higher effectiveness of phage therapy in this anti-inflammatory aspect (Liu et al., 2024).

Capparelli et al (Capparelli et al., 2007). used phage M^Sa^ in BALB/c mice infected with *S. aureus* (including MRSA strains) intravenously. Direct application of phages by the same route resulted in a 100% survival rate when the highest dose was used (10^9^ PFU/mouse). Moreover, the administration of phages 10 days after infection resulted in the clearance of bacteria in the spleens, kidneys, hearts, and blood, while they were still detected in the control group on day 20. In another study, phage therapy was applied in a clinically relevant rabbit model with fracture-related infection caused by *S. aureus* (Onsea et al., 2021). The intraoperative application of phage in saline was highly effective in preventing infection, as six out of eight animals (75%) were not infected at euthanasia (day 28), while in the control group, only one out of seven animals (14%) was infection-free. However, some difficulties were also noted in treating established staphylococcal infections. The first one is the appearance of phage-neutralizing antibodies, whose count depends on the virus administration route. The second, however, is associated with biofilm and implants, for which treatment phages were loaded in hydrogels. The low effectiveness of this solution might be due to the low titer of phage in the gel (10^7^ PFU/ml), which underlines that the phage particle carriers should allow the high concentration of virions to be gained.

Takemura-Uchiyama et al (Takemura-Uchiyama et al., 2014). investigated phage therapy in a mouse model of sepsis originating from the lungs using phage S13’. Intramuscular administration of the phage 6 hours after infection reduced the severity of the infection and saved 67% of infected mice on day 5, while only 10% of the control group survived. Importantly, the ratio did not change to the end of the experiment. Similarly, Prazak et al (Prazak et al., 2019). demonstrated that intravenous administration of a phage cocktail was as effective as teicoplanin (58 and 50% survival, respectively) in improving survival and reducing bacterial counts in the lungs of rats infected with a methicillin-resistant strain (MRSA). Although, combining antibiotics with phage therapy did not further improve outcomes. Another research showed that nebulization of phages can also reduce the number of bacteria in the lungs of MRSA pneumonia rat model (Prazak et al., 2022).

Bacteriophages may also be an effective local therapy against wounds infected with *S. aureus* biofilm. Researchers showed that in biofilm-infected wounds caused by *S. aureus* strains, after applying sharp debridement and bacteriophages, all measured wound healing parameters significantly improved and bacterial counts decreased (Seth et al., 2013). Additionally, some studies show that the application of bacteriophages in *S. aureus* infections might yield better results when combined with supplements like lactoferrin and that they do not influence the viability of skin cells (Kosznik-Kwaśnicka et al., 2023; Grzenkowicz et al., 2025; Piechowicz et al., 2025). Studies have also shown that phage therapy can be useful in the management of biofilm created on surfaces, which is important due to an increased risk of blood contamination with these bacteria during various medical procedures, such as, e.g., catheterization. Moreover, antibiotics administered on the surface after phage application are often more effective than those administered without phages (Atshan et al., 2023; Liu et al., 2024). Also, as previously established, phages are not only powerful antimicrobial factors themselves but can also be the source of antibacterial bacteriophage-derived enzymes. An example of lysin produced by phages active against *S. aureus* is Exebacase (Lysin CF-301), which exhibits an anti-biofilm effect that improves when combined with lysostaphin (Schuch et al., 2017).

### 4.6 Mycobacterium tuberculosis

Due to the ongoing rise of antibiotic-resistant strains (MDR-TB, XDR-TB), phage therapy has once again become a potentially crucial part of TB therapy. Although studies on this topic are still limited, many different approaches to the clinical applications of mycobacteriophages have already been described. Some studies focus on individual phages like D29 or DS6A (Singh et al., 2023; Yang et al., 2024), while other underline the need for a curated phage cocktail, which would help maximize the tuberculocidal effect while minimizing the chance of resistance emergence (Guerrero-Bustamante et al., 2021).

LysB, an endolysin produced by phage D29, effectively cleaves the ester bonds in the mycolic acid layer of the mycobacterial cell envelope, enabling the lysis of *M. tuberculosis in vitro* (Abouhmad et al., 2020; Singh et al., 2023). Studies show that both the phage itself and the purified LysB might be valuable additions to other phages and to rifampicin (and isoniazid), as the synergistic effect was observed *in vitro* in both cases (Guerrero-Bustamante et al., 2021; Singh et al., 2023). Although D29 shows promising results, a recent study suggests that its efficacy in agar plates doesn’t positively correlate with its efficacy in broth culture, which might lead to problems later on with using D29 on mice or more humanized models (Yang et al., 2024). This study also demonstrates phage DS6A as the better alternative, because it lysed *M. tuberculosis* both in agar plates and in liquid culture. Additionally, DS6A was tested on Mtb in primary human macrophages, which ended in a successful bacilli eradication within seven days. That result was further positively translated into a humanized mouse model treated intravenously with DS6A, which showed reduced bacterial loads in infected organs, improved pulmonary function, and weight gain compared to untreated controls. Due to the route of DS6A administration, phage distribution varied, with DS6A gathering in higher amounts in the spleen compared to the lungs, suggesting the need for alternative delivery methods to optimize lung clearance.

Aside from individual phages, researchers also explored the possibility of using various phages together to achieve the best possible clinical outcome. In a study conducted at the University of Pittsburgh, researchers have selected and tested out various phages, both the naturally lytic mycobacteriophages and particularly engineered derivatives of temperate mycobacteriophages, to finally choose the final set for the cocktail (Guerrero-Bustamante et al., 2021). Their selection criteria included broad host range, minimized risk of resistance and cross-resistance, lytic tuberculocidal activity, synergy with antibiotics, and ease of propagation. That way five phages broke forth—Adephagia Δ41Δ43, D29, Fionnbharth Δ45Δ47, Fred313_cpm Δ33, and Muddy_HRMN0157-2. However, the authors admit that this cocktail is likely to be refined before clinical evaluation, which underlines the need for more studies to be done on the final selection of the most adequate phages.

### 4.7 Anaerobic bacteria

Bacterial meningitis caused by obligatory anaerobes is a rare (<1%) but potentially severe central nervous system (CNS) infection. It requires prompt diagnosis and targeted treatment. The most isolated obligate anaerobes in meningitis are *Bacteroides fragilis, Fusobacterium necrophorum, Clostridium perfringens*, and *Cutibacterium acnes*.

*Bacteroides fragilis* typically requires specific conditions (post-surgery state, trauma, immunodeficiency) to induce bacteremia grave enough to attack the CNS (Zafar and Saier, 2021). That’s why anaerobic meningitis caused by *B. fragilis* is a rather rare occurrence, especially in adults (Feder, 1987). Nevertheless, with the rise of antibiotic resistance, alternative antimicrobial therapeutics are growing in importance, renewing researchers’ interest in possible phage solutions (Centers for Disease Control and Prevention (CDC), 2013; Merchan et al., 2016; Yekani et al., 2022). For now *B. fragilis* phages are mentioned in literature mostly as the means of monitoring the fecal contamination of water (Queralt et al., 2003), with only limited studies on possibly therapeutic phages. One study focuses on the phage vB_BfrS_23 (Tariq et al., 2020), but due to a very narrow host range, its therapeutic usefulness might be limited. Nevertheless, a more recent study characterizes GEC_vB_Bfr_UZM3 as a possible therapeutic bacteriophage against *B. fragilis*. UZM3 is a strictly lytic phage able to maintain stability for around 6 hours at body temperature and in body pH environments. Depending on the strain on which UZM3 was propagated, the phage’s host range varied from five to nine bacterial isolates out of 15, which translates into a possibly higher clinical value of UZM3 compared to vB_BfrS_23 (Bakuradze et al., 2023). Another therapeutically promising phage is vB_BfrS_VA7 (VA7), which is characterized by its virulent nature and its ability to effectively reduce *B. fragilis* cell counts in the cell culture by 2 logs (Bakuradze et al., 2021). Moreover, the same study suggests that the immunomodulating effect of VA7 (lowering the mean level of IL-8, compared to untreated cell culture) might be additionally advantageous in a potential clinical setting. Unfortunately, due to the scarcity of studies on all of the phages listed above, there is still a long way to go before any of those phages are used to treat *B. fragilis* anaerobic meningitis.

Meningitis caused by *Fusobacterium necrophorum* is also rather rare, which translates into the number of studies being published - only two relevant studies touch on the topic of *F. necrophorum* phages. The first one describes the isolation of phage FnP1, which proved effective on biovar A (5 out of 7 strains) and B (7 out of 7 strains) of *F. necrophorum*, but not on biovar C (Tamada et al., 1985). Unfortunately, that phage wasn’t studied further, so it’s hard to establish its relevance in a potential therapeutic setting. The second study, a fairly recent one, explores six novel phages - φFN37, φRTG5, φKSUM, φHugo, φPaco, and φBB (Schwarz et al., 2024). Among these phages, two (φKSUM and φBB) showed the most promise for therapeutic application against *F. necrophorum*, as both infected and suppressed growth of the *F. funduliforme* subspecies. Additionally, even though all six phages contained genes associated with lysogeny, φKSUM and φBB did not form lysogens, making them stronger candidates for potential biocontrol. To fully explore the potential of *F. necrophorum* phages as viable alternatives to antibiotics, further investigation is still needed.

Even though *Clostridium perfringens* doesn’t commonly cause meningitis, many studies explore the possible use of its phages in the clinical context due to other aspects of its pathogenicity. Most *C. perfringens* phages are explored in the context of poultry treatment, but their use in human infections might still be possible when adequate studies are undertaken. One study summarizes the twenty phages characterized more thoroughly up until 2022, with six of them used successfully to reduce necrotizing enterocolitis-associated mortality in broiler chickens by 92% and to control necrotic enteritis in them (Miller et al., 2010; Bae et al., 2021; Venhorst et al., 2022). Additionally, it was ascertained that phage phiCJ22 is highly stable under pH 5–9 and at the temperature reaching 60 °C, which makes it a viable phage for potential use in more humanized models. In another study, 32 phages were isolated, out of which five had the broadest host ranges and killed over 90% of the examined *C. perfringens* strains (Thanki et al., 2024). The last two phages worth mentioning are DCp1 and vB_CpeS_BG3P (BG3P). DCp1 was proved to be effective against *C. perfringens* biofilm, making it possibly feasible for future therapeutic use (Tang et al., 2023). BG3P, on the other hand, remains stable under pH 2–7 and temperatures up to 50 °C. It is also able to completely inhibit bacterial growth compared to untreated cultures, and produces the endolysin LysCP28, which is characterized by high antimicrobial and antibiofilm activity (Huang et al., 2022; Lu et al., 2023). Despite the promising amount of possibly useful phages, more studies need to be conducted to test their feasibility on humanized models.

Meningitis caused by *Cutibacterium acnes* is rare and usually associated with neurosurgical procedures, the presence of intracranial devices, or a long-standing shunt. In patients without such factors, cases are very sporadic (Prozan et al., 2020). Preclinical studies on phage therapy for *Cutibacterium acnes* currently focus primarily on dermatological applications. An important early study focused on the phage PA6, which effectively infects and destroys all 32 *Cutibacterium acnes* isolates (types I and II) used in the study (Farrar et al., 2007). Other researchers confirmed the activity of the phage PAP 1–1 against pathogen cells, with more promising results obtained when the phage was combined with bacteriocin from *Lactococcus lactis* CJNU 3001 and nisin (Han et al., 2023). Additionally, another study described phage CAP 10–3 isolated from a human acne lesion and proved the activity of its lysin in a dose-dependent manner (Kim et al., 2023). In the same year, a putative N-acetylmuramoyl-L-alanine type-2 amidase (PaAmi1) from bacteriophage PAC1 was characterized, and its peptidoglycan degradation activity was confirmed (Varotsou et al., 2023). The results of this study suggest that PaAmi1 is a new, effective antimicrobial agent and provide proof of concept that bacteriophage genomes are a rich source of antimicrobial proteins that can be further used to design novel or improved endolysins.

### 4.8 Zoonotic bacteria

*Brucella* is a zoonotic bacterium that primarily causes brucellosis but can sometimes cause more complicated conditions, such as meningitis (Mousa et al., 1986). It can affect both humans and animals, and its therapy is currently challenging. Phage therapy for *Brucella* spp. infections (brucellosis) is a very promising area but still relatively early in the research phase. Because of the intracellular development of this pathogen, different approaches using phages are considered. In a Swiss albino mouse model infected with *Brucella abortus*, it was found that intravenous administration of the phage (10^6^ PFU/mL) significantly reduced the number of bacteria in the spleen by 2 logs in the treatment group compared to the control group (Prajapati et al., 2014). In a study described by Jain et al (Jain et al., 2017), phage lysate of *B. abortus* was used for immunization of guinea pigs, resulting in satisfactory protection against the pathogen comparable to a vaccine. Moreover, transfer of immunized guinea pigs’ serum to mice also caused sufficient immunization. In another study, *Brucella* strain 19 was used as a carrier to deliver the phage inside the phagocytes where this pathogen resides during an infection (Mohan and Saxena, 2020). The authors suggest that it’s important to apply the mixture of bacteria with phages before lysis can be observed *in vitro*. Such action allows bacteria with phages to infect phagocytes and then phages to start lysing the carrier. Next, phage progeny infects and lyses *Brucella* cells from the original infection. The cattle with brucellosis were treated with 2 mL of *B. abortus* strain 19 vaccine with brucellaphage through the subcutaneous route. The use of bacteriophages resulted in an increase in the antibody levels compared to the prevaccination level. These results underline that phages, despite their inability to infect human cells directly, can be used to treat intracellular infections.

Leptospirosis is a zoonotic infection that causes more than a million human infections annually. Although leptospirosis affects many organs, the form involving the central nervous system (especially aseptic meningitis) is less frequently recognized. In one series of suspected aseptic meningitis cases, it was observed that Leptospira may be responsible for approximately 5−13% of all aseptic meningitis cases in certain regions (Bandara et al., 2021). The potential of phage therapy for leptospirosis remains underexplored. Only a few phages have been identified: vB_LbiM_E1 (LE1), vB_LbiM_LE3 (LE3), and vB_LbiM_LE4 (LE4) (Girons et al., 1990). Unfortunately, among the tested strains, they were active only against the Patoc serovar and the *L. meyeri* strain 201601301, indicating their narrow host range. Additionally, various mechanisms of phage resistance exhibited by *Leptospira*, such as the CRISPR-Cas system, type IV restriction–modification (R–M) system, effector protein PrrC, and the single-gene anti-phage system Borvo, might complicate the use of *Leptospira* phages even more (Petakh et al., 2024). Currently, phages against *Leptospira* are mostly used not due to their antibacterial activity but because of their ability to display different proteins on their structure, allowing the investigation of interactions between bacteria and cells or even modulation of antibodies (Lima et al., 2013; Mohd Ali et al., 2021).

## 5 Benefits and potential risks associated with phage therapy.

Amid the growing threat of antibiotic resistance, phage therapy presents both opportunities and challenges. One of the key benefits of phage therapy is its high specificity. It enables the treatment of challenging infections by targeting specific strains, without disturbing the natural microbiota. Other notable advantages of phage therapy are the relatively low costs of production and the high detectability rate. After all, phages against many pathogenic bacteria are easily discovered, often from sewage and other waste materials. The potential for synergistic use with antibiotics along with a variety of administration forms are also important, positive aspects of potential use. An equally essential element of phage therapy is its capacity for low-dosage use, due to the ability of phages to increase in density *in situ*. It’s also a very safe method, because phages will only increase in density if they are actively killing bacteria and do not otherwise linger long within the body. Another important fact is that the size of the phages allows them to permeate areas that are impenetrable by drug molecules. For example, they can cross the blood-brain barrier, which is crucial for the treatment of meningitis (El-Shibiny and El-Sahhar, 2017). However, phages planned to be used in meningitis should be checked toward this ability, as it may vary between entities. Additionally, other parts of the human body may still be difficult for phage application (e.g., bones, joints, deep wounds). The bone tissue is dense, and its vascularization is limited, which may impede the phages’ ability to reach the site of infection. On the other hand, in the case of burn patients or those with severe infections, phages can be delivered directly to the site of infection, e.g., through injections or specialized delivery methods such as bioactive scaffolds or hydrogels.

Although bacteria may develop resistance to phages specific to them, that does not always directly lead to resistance to other phages with a similar host range. Phages differ in the mechanisms they use to attack bacterial cells, as well as in the receptors they bind to in order to initiate infection (Lin et al., 2017). The downside of this property is the need for an identification of all of the strains causing the infection each time, making it necessary to apply phages specific to each strain. Another possible challenge is that some strains can develop resistance to whole groups of phages through changes in surface receptors, inhibition of the phage replication cycle, and removal of phages by bacterial defense systems such as the CRISPR-Cas system. However, a high frequency of mutation allows phages to co-evolve with their hosts, leading to combating the host defense. Another significant disadvantage of bacteriophage treatment is the possibility of occurrence of phage-neutralizing antibodies that will probably impede the effectiveness of combating the bacterial pathogen. A study conducted in 1987 analyzed fifty-seven cases of bacterial infections subjected to phage therapy and the production of antibodies against the applied bacteriophages (Kucharewicz-Krukowska and Slopek, 1987). This study implied the potential for the presence of such antibodies, which was confirmed by many researchers (Gembara and Dąbrowska, 2021; Kaźmierczak et al., 2021). However, it is likely that the production of these antibodies will not impact the efficiency of the therapy, given that it is a relatively slow process compared to the speed of phage activity. Further concerns involve the application of phage therapy in the treatment of intracellular bacterial infections. In such cases, phages can be used for immunization or packed within carriers, allowing them to get into the environment of infected eukaryotic cells (Harada et al., 2018). Currently phage cocktails can only be used on foods infected with intracellular bacteria to prevent the infection and possible secondary meningitis. Another drawback is that the pharmacokinetic properties of individual groups may unpredictably affect the quality and success rate of the therapy. They may exhibit different behaviors *in vivo* compared to *in vitro* conditions, which is why more studies are needed to ascertain their safety.

## 6 Summary and conclusions

Meningitis-related pathogens are currently one of the main concerns in CNS diseases, due to the growing antibiotic resistance detected in bacteria. Therefore, it is crucial to develop new antimicrobial therapies. Despite the drawbacks, phage therapy is being rediscovered as a safe method since these biological entities, devoid of any metabolic machinery, do not have affinity to eukaryotic cells. Naturally, more studies will be needed to properly ascertain specific phages’ viability. For now, it is assumed that the pharmacokinetics of phages rely on various factors, like administration routes, that should be based on the pathogen localization. In phage therapy of meningitis, intravenous administration is preferred, as much research suggests that phages may not only cross the blood-brain barrier but also simultaneously act in blood, in organs like the spleen and liver, and at the primary site of infection (El-Shibiny and El-Sahhar, 2017; Nguyen et al., 2017; Nang et al., 2023). Such administration significantly exceeds the oral application, in which phages are subjected to the action of acidity and are not always able to absorb into the circulatory system due to the interactions with the digestive elements. However, the presence of phages in different tissues may also be altered by the immune system, which often starts to produce antiphage antibodies (Łusiak-Szelachowska et al., 2025). Another factor influencing both pharmacokinetics and pharmacodynamics is the phage dose. On the one hand, a low dosage of phage may be neutralized by the host before reaching the site of infection, while a too high number of particles may result in the quick bacterial cells’ disruption, causing the release of a high LPS amount (Singh et al., 2022). Phages can also act differently, depending on the time of administration, as most research shows that delayed single-dose administration may not result in the improvement of health, implying the advantage of daily treatment. However, since phages, in contrast to antibiotics, can multiply themselves, their pharmacokinetics, pharmacodynamics, dosage, and adverse effects are some of the factors that should be examined extensively.

Lack of this information is one of the reasons why phage therapy is still not approved for commercial use in humans. Currently, only 9 phage preparations have received FDA approval and 3 have received European Food Safety Authority (EFSA) approval for the treatment of infections in companion animals (Berryhill et al., 2025). Moreover, there are also law regulations that discourage scientists from the usage of bacteriophages. In compassionate use, data about the performance of phages are not gathered and deeply analyzed in a precisely defined manner. On the other hand, in clinical trials, phages are treated as drugs. For these viruses, which can increase their amount in the host and evolve during that process, restrictions for drugs are extremely hard to achieve. Therefore, one of the proposed solutions is to create a scheme of models to test the different host-phage interactions (Berryhill et al., 2025). However, without the creation of specific regulations, even with clinical data implying high efficiency, phages may never be approved as antibacterials.

Since bacteria may also develop resistance to phages and these viruses have a different host range, it seems that phage therapy should be limited to personalized use. In such an approach, administration of phages should be considered in the hard-to-treat infections with the MDR pathogen. However, phages often act synergistically with antibiotics, so their applications from the beginning of the disease are also often considered. Thus, simultaneous action of these 2 antibacterials increases the ratio of infection recoveries but, on the other hand, increases the cost of therapy. Because in case of meningitis, empirical treatment is applied prior to the pathogen identification, antibiotics will remain the main therapy. The research shown in this paper revealed that phages could be a perfect complement in the fight against CNS infections or even used as a prevention in case of the meningitis development risk. Moreover, as an antibacterial agent, it helps not only to decrease the severity of symptoms, like other therapy additives, e.g., corticosteroids, but also acts directly against its cause. Nevertheless, to better understand the possible role of bacteriophages in fighting CNS infections, interactions of phages with standard therapies, including adjunctive ones, should also be tested. In summary the development of treatment methods based on phages can not only help in the fight with MDR infections, including pathogens causing meningitis, but also decrease the tempo of resistance development.
